# The Mechanism of Protective Action of Plant-Derived Squalane (2,6,10,15,19,23-Hexamethyltetracosane) Against UVA Radiation-Induced Apoptosis in Human Dermal Fibroblasts

**DOI:** 10.3390/antiox14070853

**Published:** 2025-07-11

**Authors:** Katarzyna Wolosik, Magda Chalecka, Gabriela Gasiewska, Jerzy Palka, Arkadiusz Surazynski

**Affiliations:** 1 Independent Cosmetology Laboratory, Medical University of Bialystok, Kilinskiego 1, 15-089 Bialystok, Poland; katarzyna.wolosik@umb.edu.pl; 2 Department of Medicinal Chemistry, Medical University of Bialystok, Kilinskiego 1, 15-089 Bialystok, Poland; magda.chalecka@umb.edu.pl (M.C.); gabriela.gasiewska@s.d.umb.edu.pl (G.G.); jerzy.palka@umb.edu.pl (J.P.)

**Keywords:** squalane, UVA radiation, oxidative stress, DNA damage, ROS, apoptosis

## Abstract

Ultraviolet A (UVA) radiation has been identified as a significant factor contributing to skin photoaging and skin diseases, operating through the excessive generation of reactive oxygen species (ROS) and the subsequent induction of DNA damage. Plant-derived antioxidants have demonstrated efficacy in mitigating UVA-induced damage; nevertheless, their instability limits their therapeutic potential. This study investigates the mechanisms of antioxidant and cytoprotective effects of squalane (Sq), a stable, plant-derived triterpene, in human dermal fibroblasts (HDFs) exposed to UVA radiation. Sq was administered at concentrations ranging from 0.005% to 0.015% prior to UVA exposure (10 J/cm^2^). It has been found that Sq counteracted UVA-induced ROS formation, decreased the level of reduced thiol groups, activated apoptosis, and inhibited DNA biosynthesis. Immunofluorescence analysis revealed that Sq suppressed the UVA-induced expression of p53, caspase-3, caspase-9, and PARP, while restoring the activity of the pro-survival p-Akt/mTOR pathway. The findings indicate that Sq exerts protective effects on UVA-induced fibroblast damage through a combination of antioxidant and anti-apoptotic mechanisms.

## 1. Introduction

Ultraviolet A (UVA) radiation—with wavelengths between 320 and 400 nm—is recognized as a major environmental factor contributing to skin aging and skin diseases. In contrast to UVB, which is known to induce direct DNA damage, UVA has been found to initiate oxidative damage through the excessive production of reactive oxygen species (ROS). These free radicals cause indirect DNA strand breaks, lipid peroxidation, protein oxidation, and mitochondrial dysfunction. Consequently, UVA triggers intrinsic apoptotic signaling cascades, with a central role of p53, caspase-3, caspase-9, and poly(ADP-ribose) polymerase-1 (PARP) [1,2,3,4].

Of particular significance is the finding that chronic exposure to UVA leads to the selection of apoptosis-resistant fibroblasts, which may contribute to photo-induced genomic instability. The underlying mechanism involves altered redox signaling, the depletion of reduced thiols, and the suppression of pro-survival pathways such as Phosphorylated Protein Kinase B (p-Akt)/Mammalian Target of Rapamycin Kinase (mTOR) [2]. Therefore, targeting redox signaling can modulate these molecular pathways.

Plant-derived antioxidants have received increased attention for their ability to mitigate UVA-induced oxidative damage and apoptotic responses. Compounds such as resveratrol, epigallocatechin gallate (EGCG), and sea buckthorn or *Amaranthus cruentus* seed oils have been shown to inhibit UV-triggered caspase activation, ROS levels, and mitochondrial dysfunction in dermal cells [5,6,7,8]. Nevertheless, a considerable number of polyphenolic compounds are characterized by instability, particularly in the presence of UV radiation, and present challenges concerning bioavailability.

Squalane (2,6,10,15,19,23-Hexamethyltetracosane), a fully saturated hydrocarbon, offers significant advantages over unsaturated antioxidants. Due to its lipophilicity, oxidative stability, and ability to integrate into cellular membranes, Sq has been widely used in dermatology for barrier protection and hydration [9]. However, its role in modulating oxidative stress responses at the molecular level remains incompletely understood. Although our previous studies have indicated some photoprotective potential [10], the present work offers the first comprehensive analysis of squalane’s ability to preserve intracellular redox homeostasis, limit thiol oxidation, and modulate intrinsic apoptotic signaling in UVA-exposed human dermal fibroblasts. These findings provide new mechanistic insights into the cytoprotective functions of Sq under UVA-induced oxidative stress conditions.

Compared to other plant-derived antioxidants such as resveratrol, EGCG, and squalene, Sq offers a unique combination of chemical stability, photostability, and dermal bioavailability. Polyphenolic compounds are susceptible to rapid degradation under UV exposure and often show limited skin penetration [11,12,13,14]. Squalene, although naturally present in skin lipids, is susceptible to peroxidation, which may compromise its efficacy in oxidative environments [9,15]. In contrast, the saturated hydrocarbon structure of squalane makes it inert and resistant to oxidative degradation, while its lipophilic nature ensures efficient membrane integration and cellular uptake. These comparative properties are summarized in Table 1, which compares key functional, biochemical, and formulation parameters of Sq, resveratrol, and EGCG. These properties suggest that Sq may overcome the limitations of conventional antioxidants and serve as a stable, bioavailable, and multifunctional protective agent in UVA-induced skin damage.

In our previous study [10], we demonstrated that low concentrations of Sq significantly improved the viability of HDFs exposed to UVA radiation. These findings suggested a cytoprotective effect associated with its antioxidant properties. However, the intracellular mechanism responsible for this protection remained unknown.

The present study was undertaken to explore the molecular mechanisms underlying the cytoprotective effect of Sq in HDFs exposed to UVA radiation. Specifically, the expression of apoptosis-related proteins (p53, caspase-3, caspase-9, PARP), ROS formation, the redox state of thiol groups, and the activation status of the p-Akt/mTOR pro-survival pathway were evaluated using cytometric, biochemical, and fluorescence imaging methods.

## 2. Materials and Methods

### 2.1. Materials

The compound used in this study was Neossance™ squalane (Sq) supplied by Aprinnova, LLC Emeryville, CA, USA. This compound is a highly purified plant-derived hydrocarbon obtained from renewable sugarcane feedstock and produced in compliance with Bonsucro sustainability standards. According to the manufacturer, this product is identified by the IUPAC name 2,6,10,15,19,23-Hexamethyltetracosane, with a minimum purity of 92%. It is a clear, colorless and almost odorless liquid with a density in the range of 0.806–0.811 g/cm^3^. Its iodine value does not exceed 2 g I_2_/100 g and its acid value remains below 0.5 mg KOH/g oil, confirming its chemical stability and low degree of unsaturation.

### 2.2. Methods

#### 2.2.1. Cell Culture, Squalane Treatment, and UVA Irradiation Protocol

Primary human dermal fibroblasts (HDF) were purchased from the American Type Culture Collection (ATCC, Manassas, VA, USA). Cells were cultured in Dulbecco’s Modified Eagle’s Medium (DMEM; PAN™ Biotech, Aidenbach, Germany) supplemented with 10% fetal bovine serum (FBS; Gibco, Waltham, MA, USA), 50 U/mL penicillin (Pen; Gibco, Waltham, MA, USA), and 50 μg/mL streptomycin (Strep; Gibco, Waltham, MA, USA). Cultures were maintained at 37 °C in a humidified atmosphere containing 5% CO_2_. Cells were seeded in 100 mm culture dishes with 10 mL of complete medium, which was refreshed every 2–3 days. Experiments were performed with cells between passages 8 and 10. For specific assays with the test compound, cells were incubated in DMEM without FBS and antibiotics.

HDFs were cultured in 100 mm dishes to full confluence and treated with Sq at concentrations of 0.005%, 0.01%, and 0.015% in complete DMEM. After incubation for 30 min at 37 °C, the Sq-containing medium was removed, and the cells were gently washed with cold PBS (4 °C). The fibroblasts were then exposed to UVA radiation at a dose of 10 J/cm^2^ using a Bio-Link BLX 365 crosslinker (Vilber Lourmat, Eberhardzell, Germany), which emits long-wave UV light at 365 nm. Irradiation was performed in 1 mL of cold PBS. Immediately after irradiation, the PBS was replaced with fresh DMEM, and the cells were incubated for a further 24 h under standard culture conditions. The control cells were incubated under the same conditions in PBS without exposure to UVA radiation or treatment with Sq. The selected Sq concentrations and UVA dose were based on previous MTT assay results indicating their efficacy in preserving cell viability under oxidative stress conditions [5,10]. In our earlier study [10], we also demonstrated that concentrations above 0.015% (specifically 0.02% and 0.025%) reduced fibroblast viability to 78% and 74% of control values, respectively. Therefore, only non-cytotoxic concentrations (0.005–0.015%) were selected for the present work to ensure the reliability of the mechanistic analysis and to avoid confounding effects related to viability.

#### 2.2.2. Reactive Oxygen Species (ROS) Formation

HDFs were cultured in black 96-well plates at a density of 1000 cells per well. Once full confluence was reached, the culture medium was removed, and the cells were gently washed with PBS. Then, 100 µL of complete DMEM containing Sq at a concentration of 0.005%, 0.01%, or 0.015% was added to each well. After 30 min of incubation at 37 °C, the medium was discarded, and the cells were washed with cold PBS (4 °C). The fibroblasts were then exposed to UVA radiation at a total dose of 10 J/cm^2^ (λ = 365 nm) in 100 µL of cold PBS (4 °C). Immediately after irradiation, the PBS was replaced with fresh DMEM, and the cells were incubated for a further 4 h under standard culture conditions (37 °C, 5% CO_2_). The control cells were incubated under the same conditions in PBS without exposure to UVA radiation or treatment with Sq.

To assess intracellular ROS levels, 0.5 µM 2′,7′-dichlorodihydrofluorescein diacetate (DCFDA) was added to each well, and the plates were incubated for 15 min at 37 °C in a 5% CO_2_ atmosphere. DCFDA is a non-fluorescent compound that is converted to the highly fluorescent 2′,7′-dichlorofluorescein (DCF) upon oxidation by ROS within the cell. After incubation, the DCFDA-containing medium was removed, and the cells were washed twice with pre-warmed PBS. Then, 100 µL of PBS was added to each well. Fluorescence imaging was performed using a BD Pathway 855 Bioimaging System equipped with an environmental control chamber set at 37 °C and 5% CO_2_. Excitation and emission wavelengths were set at 488 nm and 521 nm, respectively. Fluorescence intensity correlates with the amount of ROS present in the cells, allowing a quantitative assessment of the level of oxidative stress.

#### 2.2.3. Apoptosis Cytometric Assay

HDFs were seeded in 6-well plates at an initial density of 3 × 10^5^ cells per well. Once full confluence was reached, the culture medium was removed, and the cells were gently washed with PBS. Then, 2 mL of complete DMEM containing Sq at a concentration of 0.005%, 0.01%, and 0.015% was added to each well. After 30 min of incubation at 37 °C, the medium was discarded, and the cells were washed with cold PBS (4 °C). The fibroblasts were then exposed to UVA radiation at a total dose of 10 J/cm^2^ (λ = 365 nm) in 1 mL cold PBS (4 °C). Immediately after irradiation, the PBS was replaced with fresh DMEM, and the cells were incubated for a further 24 h under standard culture conditions (37 °C, 5% CO_2_). The control cells were incubated in the same conditions in PBS without exposure to UVA radiation or treatment with Sq.

After incubation, both adherent and floating cells were collected by trypsinization and transferred to 1.5 mL microcentrifuge tubes. The cell suspensions were centrifuged at 800× *g* for 5 min at room temperature. The supernatants were discarded, and the cell pellets were resuspended in 100 μL Annexin V binding buffer (ChemoMetec, Lillerød, Denmark). To each sample, 2 μL of annexin V-CF488A conjugate (Biotium, Fremont, CA, USA) and 2 μL of Hoechst 33342 solution (final concentration: 10 μg/mL) were added. The mixtures were gently pipetted to ensure thorough mixing and incubated at 37 °C for 15 min in the dark.

After incubation, the cells were centrifuged at 400× *g* for 5 min at room temperature. The supernatants were removed, and the cell pellets were resuspended in 100 μL Annexin V binding buffer supplemented with 10 μg/mL propidium iodide (PI). Immediately thereafter, 30 μL of each cell suspension was loaded into the chambers of an NC-Slide A2™ (ChemoMetec, Denmark) and analyzed using the NucleoCounter^®^ NC-3000™ fluorescence image cytometer (ChemoMetec, Denmark). Cells with low fluorescence intensities for both PI (PI-negative) and Annexin V were classified as viable, living cells. Cells with high PI fluorescence (PI-positive) but low Annexin V fluorescence were identified as early apoptotic cells. In contrast, cells with high fluorescence intensities for both PI and Annexin V were considered late apoptotic or dead. Data collection and analysis were performed using the NucleoView™ software version 2.4. (ChemoMetec, Denmark), which allows for the quantification of apoptotic cell populations.

#### 2.2.4. Free Thiol Groups Assay

HDFs were seeded in 6-well plates at an initial density of 3 × 10^5^ cells per well. Once full confluence was reached, the culture medium was removed, and the cells were gently washed with phosphate-buffered saline (PBS). Then, 2 mL of complete DMEM containing Sq at a concentration of 0.005%, 0.01%, and 0.015% was added to each well. After 30 min of incubation at 37 °C, the medium was discarded, and the cells were washed with cold PBS (4 °C). The fibroblasts were then exposed to UVA radiation at a total dose of 10 J/cm^2^ (λ = 365 nm) in 1 mL of cold PBS (4 °C). Immediately after irradiation, the PBS was replaced with fresh DMEM, and the cells were incubated for a further 24 h under standard culture conditions (37 °C, 5% CO_2_). The control cells were incubated under the same conditions in PBS without exposure to UVA radiation or treatment with Sq.

After incubation, both adherent and floating cells were collected by trypsinization and transferred to 1.5 mL microcentrifuge tubes. The cell suspensions were centrifuged at 1600× *g* for 10 min at room temperature. The supernatants were discarded, and the cell pellets were resuspended in 190 μL PBS. To each sample, 10 μL of Solution 5 (ChemoMetec, Denmark) containing VitaBright-48™ (VB-48™), acridine orange (AO), and propidium iodide (PI) was added. The mixtures were gently pipetted to ensure thorough mixing. Subsequently, 10 μL of each stained cell suspension was loaded into the chambers of an 8-chamber NC-Slide A8™ (ChemoMetec, Denmark). Slides were analyzed using the NucleoCounter^®^ NC-3000™ fluorescence image cytometer (ChemoMetec, Denmark) using the Vitality Assay protocol. This assay allows the detection of intracellular free thiol groups, primarily reduced glutathione (GSH), at the single cell level. VB-48™ is a thiol-reactive fluorescent probe that emits fluorescence upon binding to free thiol groups, allowing the assessment of cellular redox status and vitality.

During the analysis, cells with low PI fluorescence (PI-negative) and high VB-48™ fluorescence were considered viable with high intracellular thiol levels. In contrast, cells with high PI fluorescence (PI-positive) were indicated to have compromised membrane integrity, a characteristic of dead or late apoptotic cells. Fluorescence intensity data were processed using the NucleoView™ software (ChemoMetec, Denmark), which provides the quantitative assessments of thiol levels and cell viability.

#### 2.2.5. DNA Biosynthesis Assay

HDFs were seeded in 6-well plates at an initial density of 3 × 10^5^ cells per well. Once full confluence was reached, the culture medium was removed, and the cells were gently washed with phosphate-buffered saline (PBS). Then, 2 mL of complete DMEM containing squalane at a concentration of 0.005%, 0.01%, and 0.015% was added to each well. After 30 min of incubation at 37 °C, the medium was discarded, and the cells were washed with cold PBS (4 °C). The fibroblasts were then exposed to UVA radiation at a total dose of 10 J/cm^2^ (λ = 365 nm) in 1 mL of cold PBS (4 °C). Immediately after irradiation, the PBS was replaced with fresh DMEM, and the cells were incubated for a further 24 h under standard culture conditions (37 °C, 5% CO_2_). The control cells were incubated under the same conditions in PBS without exposure to UVA radiation or treatment with Sq.

To assess DNA synthesis, 10 μL of [methyl-^3^H]thymidine (0.5 μCi/mL; PerkinElmer, Waltham, MA, USA) was added to each well immediately after UVA exposure. Cells were incubated for 24 h at 37 °C in a 5% CO_2_ atmosphere. After incubation, the medium was removed, and the cells were washed twice with cold PBS to remove unincorporated thymidine. The radioactivity was measured using a Tri-Carb 2810 TR liquid scintillation analyzer (PerkinElmer, Waltham, MA, USA). The amount of [^3^H]-thymidine incorporated into DNA, expressed as disintegrations per minute (dpm), reflects the rate of DNA synthesis and hence cell proliferation. The dpm values obtained from treated samples were compared with those of the control group to determine the effect of Sq and UVA exposure on DNA biosynthesis.

#### 2.2.6. Immunofluorescence Staining and Confocal Microscopy

HDFs were cultured in black 96-well plates to full confluence. The cells were then treated with Sq and exposed to UVA irradiation. The control cells were incubated under the same conditions in PBS without exposure to UVA radiation or treatment with Sq. After 24 h of incubation, the culture medium was removed, and the cells were fixed with a 3.7% formaldehyde solution for 10 min at room temperature. After fixation, the wells were washed once with 100 µL phosphate-buffered saline (PBS).

Permeabilization was performed with a 0.1% Triton X-100 solution for 10 min at room temperature. After permeabilization, the wells were washed twice with PBS. To block non-specific binding sites, 3% FBS in PBS was added to each well and incubated for 30 min at room temperature. The blocking solution was then removed, and 50 µL of primary antibody diluted 1:50 in 3% FBS was added to each well. Plates were incubated for 1 h at room temperature. After the primary antibody incubation, the wells were washed three times with PBS. Then, 50 µL of secondary antibody diluted 1:1000 in 3% FBS was added to each well, and the plates were incubated for 1 h at room temperature in the dark. After the secondary antibody incubation, the wells were washed three times with PBS. For nuclear staining, 100 µL of PBS containing 2 µg/mL Hoechst 33342 was added to each well. Imaging was performed using a BD Pathway 855 confocal laser scanning microscope (Becton Dickinson, Franklin Lakes, NJ, USA) equipped with the AttoVision software version 1.6.

#### 2.2.7. Western Blot Analysis

Human dermal fibroblasts (HDF) were seeded into 100 mm culture dishes at a density of 2 × 10^6^ cells per dish. Once the cells had reached full confluence, they were subjected to UVA irradiation and Sq treatment, as outlined in Section 2.2.1. After a 24 h post-treatment incubation, cellular proteins were extracted using a lysis buffer containing protease and phosphatase inhibitors. Total protein concentration was quantified using the Lowry method [18]. Equal amounts of protein were separated by SDS-PAGE following the Laemmli protocol [19]. Subsequently, the proteins were transferred onto 0.2 µm nitrocellulose membranes using a wet transfer system (Trans-Blot, Bio-Rad, Hercules, CA, USA), at a current of 200 mA for three hours at 4–8 °C in Towbin buffer (25 mM Tris, 192 mM glycine, 20% methanol, 0.025–0.1% SDS, pH 8.3). The membranes were then blocked for one hour at room temperature in 5% non-fat dry milk (NFDM) dissolved in TBS-T (20 mM Tris, 150 mM NaCl, and 0.1% Tween^®^ 20). The blots were then incubated overnight at 4 °C with primary antibodies diluted 1:1000 in TBS-T. After washing, the membranes were incubated for one hour at room temperature with HRP-conjugated secondary antibodies diluted 1:3000 in 5% NFDM. Protein bands were visualized using a chemiluminescence detection system (BioSpectrum Imaging System, UVP, Ultra-Violet Products Ltd., Cambridge, UK) after additional washes with TBS-T.

#### 2.2.8. Antibodies

For immunofluorescence staining, the used primary antibodies, diluted 1:1000, are as follows: PARP Rabbit Antibody (cleaved), caspase-3 Rabbit Antibody, caspase-9 Rabbit Antibody, pAKT Rabbit Antibody, mTOR Rabbit Antibody, p53 Mouse Antibody, and β-Actin Mouse Antibody; all antibodies were purchased from Becton Dickinson (Franklin Lakes, NJ, USA). Secondary HRP-conjugated antibodies, diluted 1:3000, were from Sigma Aldrich (Saint Louis, MO, USA).

#### 2.2.9. Statistical Analysis

Statistical analysis was applied only to the DNA biosynthesis assay results. Data are expressed as the mean ± standard error of the mean (SEM) from at least three independent experiments (*n* ≥ 3), performed in triplicate. Statistical analysis was conducted using Statistica 13.3 (StatSoft, Kraków, Poland). The graph was created using Microsoft Excel 2019 (Microsoft Corporation, Redmond, WA, USA), and the standard error of the mean (SEM) was automatically calculated using Excel’s built-in chart tools. One-way analysis of variance (ANOVA), followed by Dunnett’s post hoc test, was used to compare each experimental group to the control. A *p*-value of less than 0.05 (*p* < 0.05) was considered statistically significant. Statistical results are shown in Table 2.

## 3. Results and Discussion

### 3.1. Reactive Oxygen Species (ROS) Formation

**Figure 1:**
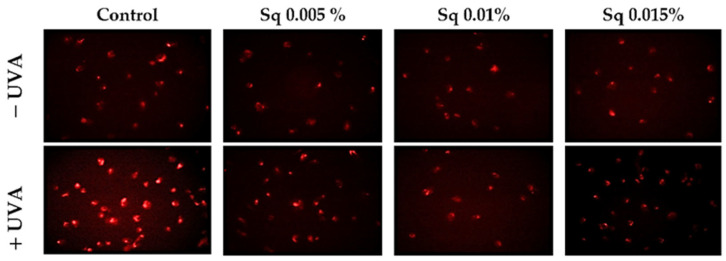
ROS generation was visualized by fluorescence in UVA-irradiated HDFs treated with Sq at concentrations of 0.005%, 0.01%, and 0.015% compared to control. The red fluorescence intensity indicates the level of ROS generated. Images were captured at 20× magnification.

UVA radiation exerts a cytotoxic effect on HDFs, primarily through the enhanced generation of ROS. Elevated ROS levels have been shown to have a detrimental effect on key cellular processes and to trigger pathways that result in cell death. This process is known as apoptosis, and it ultimately leads to cellular damage. The objective of this study was to investigate the mechanisms of antioxidant and cytoprotective capacity of Sq. The effects of Sq were evaluated at concentrations of 0.005%, 0.01%, and 0.015% in UVA-exposed HDFs. It has been found that exposure of HDFs to UVA significantly increases intracellular ROS levels. Treatment with 0.005% Sq resulted in a reduction in ROS production, while more pronounced reductions were observed at concentrations of 0.01% and 0.015% (Figure 1). This dose-dependent effect suggests that Sq effectively mitigates UVA-induced oxidative stress, potentially preserving fibroblast viability and function.

These results are consistent with those of previous studies. For instance, Gegotek et al. [6] demonstrated that the treatment of UVA-irradiated fibroblasts with sea buckthorn seed oil reduced ROS generation by approximately 25%, attributing the effect to the oil’s ability to counteract UVA-induced redox imbalance. In addition, in our earlier research, we demonstrated that *Amaranthus cruentus* seed oil exhibited potent antioxidant properties by counteracting UVA-induced oxidative stress and supporting redox homeostasis in HDFs [5]. *Amaranthus cruentus* seed oil contains significant amounts of squalene, a naturally occurring unsaturated triterpene hydrocarbon with documented antioxidant activity [10]. Squalene is the unsaturated precursor of Sq (the fully hydrogenated and more stable saturated form) that is used in the present study. The structural similarity between these compounds suggests that the antioxidant activity observed in both *Amaranthus cruentus* seed oil and pure Sq may share a common molecular mechanism. The present findings suggest that Sq can effectively reduce UVA-induced ROS production in fibroblasts, underscoring its potential as a protective agent in cases of oxidative stress-related skin damage.

### 3.2. DNA Damage and PARP Activation

**Figure 2:**
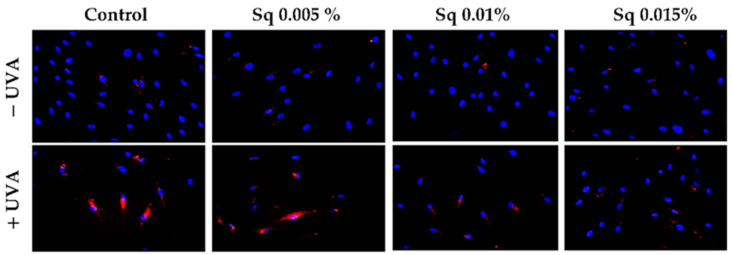
PARP protein expression visualized by the immunofluorescence staining of UVA-irradiated HDFs in the presence of Sq at concentrations of 0.005%, 0.01%, and 0.015% vs. the control. Blue staining indicates the nuclei, and red staining represents PARP protein expression. The images were obtained at 20× magnification.

The exposure of HDFs to UVA contributes to the redox imbalance and oxidative modifications of nucleic acids, proteins, and lipids [20]. A significant consequence of this process is the induction of DNA damage, encompassing single- and double-strand breaks, which, in turn, activate the cellular DNA damage response (DDR) [21]. A pivotal constituent of the DDR is poly(ADP-ribose) polymerase-1 (PARP-1), a nuclear enzyme that detects DNA strand breaks and facilitates repair through the process of ADP-ribosylation of nuclear proteins [3,22]. However, the excessive or sustained activation of PARP-1 in response to severe oxidative stress has been demonstrated to deplete NAD+ and ATP stores, ultimately leading to an energy crisis and programmed cell death. In this particular context, PARP-1 fulfils dual functions: it acts as a sensor of genotoxic stress and as a mediator of apoptosis [23]. In order to assess the potential of Sq to influence DNA integrity under conditions of UVA-induced oxidative stress, we examined PARP expression in UVA-irradiated HDF cells treated with Sq. Immunofluorescence analysis revealed an increase in PARP signal following UVA exposure. This increase was mitigated in a concentration-dependent manner by Sq, with the substantial reduction observed at 0.015% (Figure 2). The results of this study suggest that Sq has the potential to reduce UVA-induced oxidative stress and limit DNA damage, thereby indirectly modulating PARP activation.

**Figure 3:**
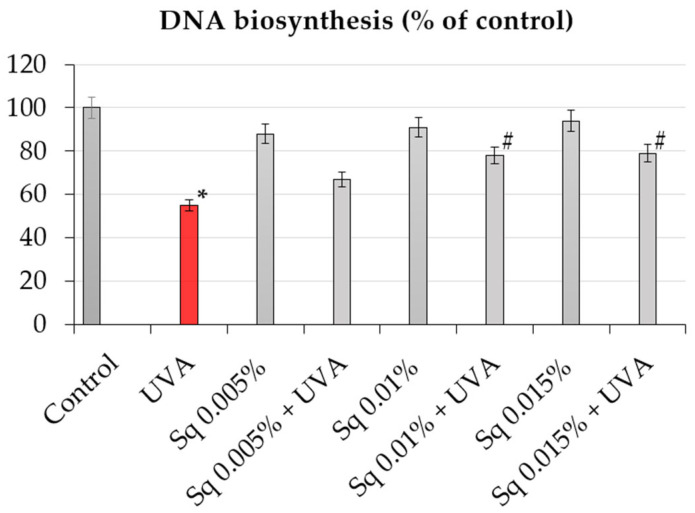
DNA biosynthesis (% of control) in UVA-irradiated HDF cells in the presence of Sq at concentrations of 0.005%, 0.01%, and 0.015%. Data represent the mean ± SEM from at least three independent experiments (n ≥ 3). Statistical analysis was performed using one-way ANOVA followed by Tukey’s post hoc test. A *p* < 0.05 was considered statistically significant. * indicates a significant difference vs. the control group; # indicates a significant difference vs. the UVA-only group (red bar).

Since DNA biosynthesis is a critical marker of cell proliferation and genomic stability, we further investigated whether Sq affects DNA synthesis following UVA-induced oxidative stress. The incorporation of [^3^H]-thymidine into DNA was used to quantify DNA synthesis in HDFs under different treatment conditions. It was found that exposure of HDFs to UVA resulted in a marked decrease in DNA biosynthesis, as detected by a decrease in [^3^H]-thymidine incorporation into DNA to about 55% of the control value (Table 2, Figure 3). However, treatment with Sq reversed this effect in a dose-dependent manner. At 0.015%, Sq restored DNA synthesis to almost 80% of the control level, demonstrating a protective effect against UVA-mediated proliferation arrest. These results support the hypothesis that Sq preserves DNA synthesis supposedly by reducing ROS accumulation and DNA damage. The observed downregulation of PARP expression and the accompanying normalization of DNA biosynthesis suggest a mechanistic link between the antioxidant activity of Sq and the maintenance of genomic function under oxidative stress [24].

In consideration of the findings, it can be concluded that UVA-induced oxidative stress initiates a series of events including ROS overproduction, impaired DNA biosynthesis, DNA damage, the activation of PARP, and decreased cell viability. It has been demonstrated that Sq interrupts this cascade by lowering ROS levels, reducing DNA damage, limiting PARP activation, and restoring DNA synthesis. Therefore, it is suggested that the antioxidant effect of Sq, particularly at concentrations of 0.01% and 0.015%, acts by neutralizing ROS. Consistent with previous reports [5,6], the present findings underscore the significance of plant-derived triterpenes in skin photoprotection.

### 3.3. Reduced Thiol Levels and Apoptosis

A critical downstream effect of UVA-induced oxidative stress is the disruption of cellular redox homeostasis, leading to the oxidation of thiol groups in proteins containing cysteine, e.g., glutathione (GSH). Under normal conditions, thiol groups exist predominantly in the reduced state, forming a buffering system that maintains structural protein integrity and regulates redox-sensitive signaling. Oxidative conditions, such as those induced by UVA radiation, disrupt this balance, leading to thiol oxidation, glutathione depletion, and the activation of programmed cell death pathways, primarily apoptosis, as confirmed in our model by flow cytometric analysis [5,25].

**Figure 4:**
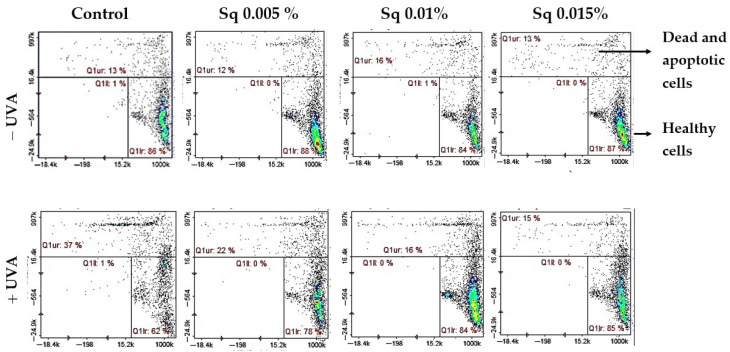
Cytometric analysis of reduced thiol levels in HDFs exposed to UVA radiation and treated with Sq at concentrations of 0.005%, 0.01%, and 0.015%. Cells were stained using the fluorescent dyes propidium iodide (PI) and VB-48^™^. Quadrants are defined as follows: lower right (Q1lr) represents healthy cells with preserved reduced thiol levels (VB-48™-positive, PI-negative); upper right (Q1ur) shows dead or apoptotic cells with diminished thiol content (PI-positive); and lower left (Q1ll) contains unstained or debris-like events. In the control group (non-irradiated cells), most cells (86%) were located in the Q1lr quadrant, indicating high levels of reduced thiols and overall cell viability. After UVA exposure, the number of cells in this quadrant dropped significantly to 62%, reflecting oxidative damage and thiol depletion. At the same time, the number of cells in the Q1ur quadrant—representing damaged or dying cells—increased to 37%. Treatment with Sq effectively counteracted these changes in a dose-dependent manner: it increased the proportion of healthy, thiol-positive cells in Q1lr (to 78–85%) and reduced the number of damaged cells in Q1ur (to 15–22%).

We found that UVA significantly reduced the level of reduced thiols in HDFs (to 62% of control), indicating redox imbalance and oxidative damage to protein structures. Importantly, treatment with Sq effectively prevented this depletion in a dose-dependent manner, restoring reduced thiol levels to 78–85% of control. These results suggest that Sq maintains the intracellular redox environment, thereby helping to preserve cellular function under conditions of oxidative stress (Figure 4).

**Figure 5:**
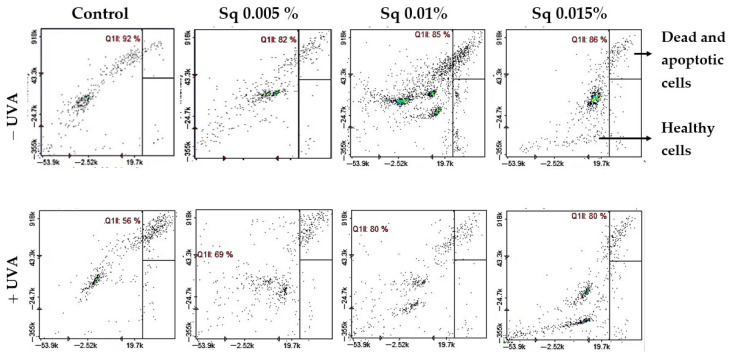
Cytometric analysis of apoptosis in HDFs exposed to UVA radiation and treated with Sq at concentrations of 0.005%, 0.01%, and 0.015%. Cells were stained with propidium iodide (PI) and Annexin V fluorescent dyes. Quadrant Q1ll contains viable, non-apoptotic cells that are characterized by negative staining for both Annexin V and PI. This indicates preserved membrane integrity and the absence of early or late apoptotic features. In the control group (non-irradiated cells), the majority of events (92%) were located in quadrant Q1ll, indicating high cell viability. UVA exposure led to a decrease in this population, reducing viable cells to 56%, indicating enhanced apoptosis. Treatment with Sq counteracted this effect in a dose-dependent manner, increasing the proportion of healthy cells in Q1ll to 69%, 80%, and 80% for Sq concentrations of 0.005%, 0.01%, and 0.015%, respectively.

This redox protection correlated with increased cell viability. Flow cytometric analysis revealed that UVA-irradiated HDFs showed a significant reduction in survival (to 56% of control), with a corresponding rise in apoptosis. Conversely, Sq-treated HDFs maintained viability at 85–86% in non-irradiated conditions and demonstrated enhanced survival following UVA exposure—up to 69%, 80%, and 80% at Sq concentrations of 0.005%, 0.01%, and 0.015%, respectively (Figure 5). These data confirm that Sq mitigates UVA-induced cell death through cytoprotective and anti-apoptotic mechanisms. Although this study assessed apoptotic cell death quantitatively using the Annexin V protocol, future experiments may benefit from complementary histological staining techniques.

It is interesting to note that the effect of Sq does not appear to promote the uncontrolled proliferation or suppress apoptosis. Rather, it selectively modulates the redox-sensitive upstream signals, leading to apoptotic commitment. This distinction assumes particular relevance in the context of skin cancer prevention, where chronic oxidative stress has been demonstrated to promote DNA mutations, premature senescence, and the establishment of a cancer-promoting microenvironment [26].

Although the present study focused on dermal fibroblasts, the effects of UVA-induced oxidative stress extend to other epidermal cells, such as keratinocytes and melanocytes, as well as immune cells like Langerhans cells, contributing to persistent pigment darkening, immune dysregulation, and mutagenesis [1]. UVA1-induced pigmentation is not photoprotective and has been associated with 8-oxoGua and CPD formation, particularly in the basal epidermal layer. Additionally, UVA exposure alters Langerhans cell morphology and density via ROS-dependent mechanisms, potentially weakening epidermal immunity and promoting a microenvironment conducive to tumor formation [27]. Given the emerging role of oxidative damage in early photo carcinogenesis, Sq’s protective role in maintaining redox homeostasis in fibroblasts may also hold relevance for other epidermal cell types exposed to UVA radiation.

Of particular concern is the basal layer of the epidermis, where UVA-induced oxidative DNA damage accumulates in stem and progenitor keratinocytes—cells recognized as the origin of many skin cancers. DNA mutations induced by UVA have been identified in transformed keratinocytes from squamous cell carcinoma (SCC) and premalignant solar keratosis lesions in nearly equal proportions, which highlights UVA’s substantial contribution to early skin carcinogenesis [28,29].

Apoptosis, a crucial component of cellular homeostasis, functions as a barrier to transformation. However, persistent oxidative stress has been observed to exert a paradoxical effect, enhancing the risk of skin cancer by impeding regenerative capacity and fostering chronic inflammation [30]. It has been demonstrated that Sq contributes to dermal photoprotection by maintaining redox homeostasis and modulating apoptosis in a balanced manner [16,17]. Whether Sq may play a role in delaying or preventing photocarcinogenic changes remains to be established.

### 3.4. Mitochondrial Apoptotic Pathway

**Figure 6:**
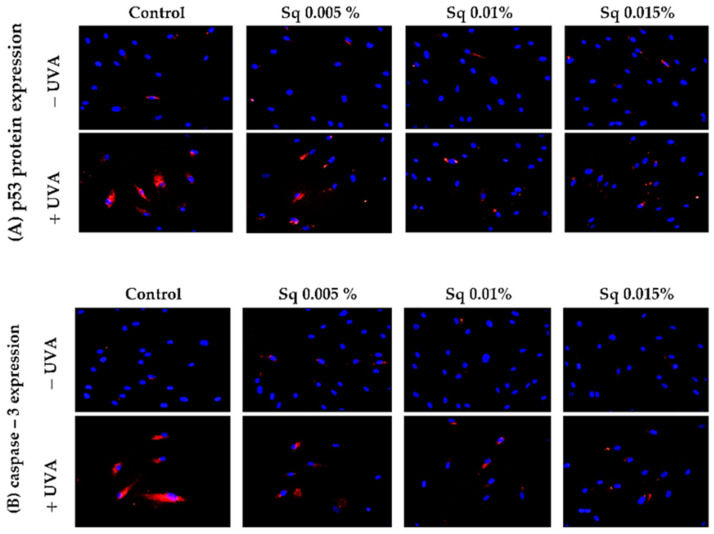
p53 (**A**) and caspase-3 (**B**) expression visualized by the immunofluorescence staining of UVA-irradiated HDFs in the presence of Sq at concentrations of 0.005%, 0.01%, and 0.015% vs. the control. Blue staining indicates the nuclei, and red staining represents protein expression. The images were obtained at 20× magnification.

**Figure 7:**
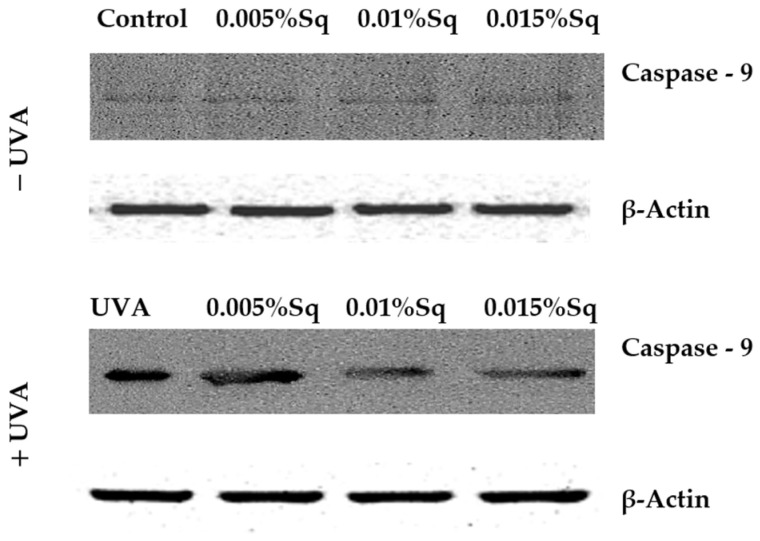
Western blot analysis for caspase-9 expression with β-Actin as a loading control for UVA-irradiated HDFs in the presence of Sq at concentrations of 0.005%, 0.01%, and 0.015% vs. the control.

Following the previous observations of reduced cell viability and enhanced apoptosis following UVA exposure, the present study has confirmed that UVA radiation at a dose of 10 J/cm^2^ induces apoptosis in HDFs. Flow cytometric analysis demonstrated a significant decrease in cell survival, which was partially reversed by Sq treatment. To further clarify the molecular mechanism, markers of the mitochondrial apoptotic pathway were examined. Immunofluorescence analysis revealed a significant increase in the expression of the tumor suppressor protein p53 (Figure 6A) and caspase-3 (Figure 6B) in UVA-irradiated HDFs, confirming the activation of apoptosis via the intrinsic (mitochondrial) pathway. To further validate this finding, we assessed the activation of caspase-9 using Western blotting, which confirmed the increased expression of its cleaved form in irradiated cells and its attenuation upon Sq treatment (Figure 7). Sq treatment was shown to reduce the UVA-induced expression of both p53 and caspase-3 in a concentration-dependent manner, with the most notable effect observed at 0.015%. The findings suggest that Sq modulates mitochondrial apoptotic signaling to promote fibroblast survival.

**Figure 8:**
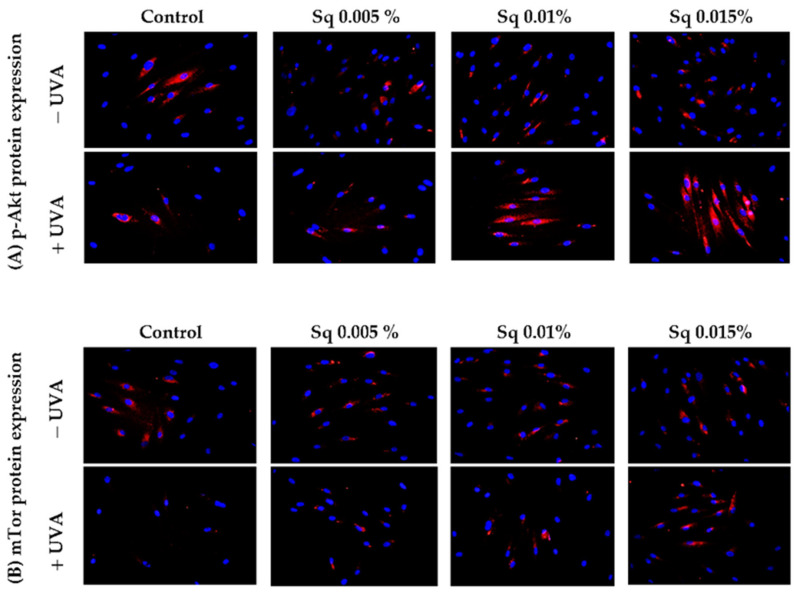
p-Akt (**A**) and mTOR (**B**) expression visualized by the immunofluorescence staining of UVA-irradiated HDFs in the presence of Sq at concentrations of 0.005%, 0.01%, and 0.015% vs. the control. Blue staining indicates the nuclei, and red staining represents protein expression. The images were obtained at 20× magnification.

A similar study was conducted to evaluate the function of Akt/mTOR proteins, pivotal pro-survival factors, in UVA-irradiated HDFs. Chronic exposure of cells to UVA has been demonstrated to inhibit the Akt/mTOR signaling, a key regulator of various physiological processes including cell growth, metabolism, and resistance to stress-induced apoptosis. As previously indicated [2], the interplay between p-Akt/mTOR and p53 is pivotal in determining cell fate in response to UVA stress. The results of the present study demonstrate that, in UVA-irradiated HDFs, the expression of phosphorylated p-Akt and mTOR was significantly reduced, suggesting the impairment of pro-survival signaling (Figure 8A,B). However, treatment with Sq at concentrations of 0.01% and 0.015% effectively restored the expression of both p-Akt and mTOR to control levels.

Protective effects on the Akt/mTOR pathway in UVA-irradiated fibroblasts were previously demonstrated for *Amaranthus cruentus* seed oil [5]. This highlights potential of Sq as a key modulator of cell survival signaling. Through this dual mechanism—the suppression of mitochondrial apoptosis and the activation of survival pathways—Sq emerges as a potent protective agent that may contribute to the preservation of dermal tissue homeostasis under conditions of UVA exposure. Taken together, these findings further support the proposed model in which Sq intervenes at multiple levels of the UVA-induced damage cascade: from limiting ROS generation and preserving redox balance to preventing DNA damage and PARP activation to restoring proliferative capacity, thus shifting the balance between apoptosis and survival towards regeneration and tissue preservation.

## 4. Conclusions

The present study demonstrates that plant-derived squalane exhibits strong antioxidant and anti-apoptotic activity in UVA-irradiated human dermal fibroblasts. Squalane significantly reduces ROS levels, preserves redox-sensitive thiol groups, limits DNA damage and poly(ADP-ribose) polymerase (PARP) activation, and restores DNA biosynthesis and the activity of the pro-survival p-Akt/mTOR pathway. While the protective properties of Sq have previously been suggested, this study is the first to comprehensively confirm its cytoprotective role through redox regulation and modulation of intrinsic apoptotic signaling.

We validated our immunofluorescence observations using Western blot analysis to examine caspase-9 activation, which supports the involvement of the mitochondrial pathway in Sq-mediated protection. The observed upregulation of p53 and caspase-3 proteins validates the anti-apoptotic effect of Sq in the context of UVA-induced oxidative injury.

These findings suggest that Sq is a biologically active compound that can protect dermal cells from UVA-induced oxidative stress and cell death. Given UVA’s broader role in damaging other skin cell types, such as keratinocytes, melanocytes, and Langerhans cells, through ROS generation and DNA damage, the redox-modulating properties of Sq may have wider dermatological implications.

This study provides a mechanistic basis for considering Sq as a multifunctional ingredient in anti-aging and photoprotective skincare formulations. Further research involving epidermal cell lines and in vivo models is recommended to confirm its potential in preventing photoaging and early photocarcinogenic changes.

## Figures and Tables

**Table 1:** Comparative physicochemical and functional characteristics of squalane, resveratrol, and EGCG.

	Squalane [10,15,16,17]	Resveratrol [13]	EGCG [8]
Parameter			
Antioxidant efficacy	Moderate to high	High	High
Photostability under UVA	Resistant to photodegradation	Rapid photodegradation	UV-labile
Susceptibility to lipid peroxidation	None (saturated molecule)	Minimal	Minimal
Skin penetration potential	High (lipophilic, membrane-integrating)	Moderate	Low
Stability in formulations	High (chemically inert, non-oxidizable)	Limited (oxidation-prone)	Low (unstable in aqueous systems)
Mechanism of action	Redox modulation, DNA protection	MAPK/NF-κB inhibition, ROS scavenging	Anti-inflammatory, MMP inhibition

**Table 2:** The dpm values of radioactive [methyl-^3^H] thymidine incorporation into DNA. Statistical analysis was performed using one-way ANOVA followed by Dunnett’s post hoc test. A *p* < 0.05 was considered statistically significant. * indicates a significant difference vs. the control group; ^#^ indicates a significant difference vs. the UVA-only group.

The dpm Values of Radioactive [methyl-^3^H] Thymidine											
Non-UVA-Treated Cells (−UVA)											
	I	II	III	I	II	III	I	II	III	Mean Value	% Value
Control	4411	4316	4363	3991	3556	3639	4039	4058	4049	4046.8	100
Sq 0.005%	3645	3769	3707	3877	3880	3879	3574	3991	3630	3574	88,334,157
Sq 0.01%	3612	3887	3608	3914	3930	3922	3491	3395	3443	36,891,111	91,179,217
Sq 0.015%	3808	3564	3532	3862	4265	3910	3688	4064	3710	38,225,556	94,477,399
**UVA-Treated Cells (+UVA)**											
UVA	1937	1833	1944	2616	2253	2326	2371	2301	2336	2213	54.69 *
Sq 0.005%	2838	2828	2833	2838	2826	2832	2542	2525	2533	27,327,778	67,542,703
Sq 0.01%	3026	3327	3295	2992	3165	3078	3113	3425	3134	31,727,778	78,417,642 ^#^
Sq 0.015%	3421	3501	3461	3228	3214	3221	2950	2959	2954	32,121,111	79,389,795 ^#^

## Data Availability

The data presented in this study are available in the main text of this article or on request from the corresponding author.

## Abbreviations

The following abbreviations are used in this manuscript:

**Sq**	Squalane
**HDFs**	Human Dermal Fibroblasts
**UVA**	Ultraviolet A Radiation
**ROS**	Reactive Oxygen Species
**PARP**	Poly (ADP-ribose) Polymerase
**GSH**	Reduced Glutathione
**PI**	Propidium Iodide
**AO**	Acridine Orange
**VB-48™**	VitaBright-48™
**DCFDA**	2′,7′-Dichlorodihydrofluorescein Diacetate
**DCF**	2′,7′-Dichlorofluorescein
**FBS**	Fetal Bovine Serum
**PBS**	Phosphate-Buffered Saline
**DMEM**	Dulbecco’s Modified Eagle’s Medium
**IGF-IR**	Insulin-Like Growth Factor I Receptor
**TGF-β**	Transforming Growth Factor Beta
**ERK1/2**	Extracellular Signal-Regulated Kinases 1/2
**COX-2**	Cyclooxygenase-2
**NF-κB**	Nuclear Factor Kappa B
**MAPKs**	Mitogen-Activated Protein Kinases
**mTOR**	Mammalian Target of Rapamycin Kinase
**p-Akt**	Phosphorylated Protein Kinase B
